# Non-Genomic Control of Dynamic *MYCN* Gene Expression in Liver Cancer

**DOI:** 10.3389/fonc.2020.618515

**Published:** 2021-04-16

**Authors:** Xian-Yang Qin, Luc Gailhouste

**Affiliations:** Liver Cancer Prevention Research Unit, RIKEN Cluster for Pioneering Research, Wako, Japan

**Keywords:** MYCN, liver cancer, microenvironment, inflammation, plasticity, lipid desaturation, endoplasmic reticulum stress, miRNA

## Abstract

Upregulated *MYCN* gene expression is restricted to specialized cell populations such as EpCAM^+^ cancer stem cells in liver cancer, regardless of DNA amplification and mutation. Here, we reviewed the role of *MYCN* gene expression in liver homeostasis, regeneration, and tumorigenesis, and discussed the potential non-genomic mechanisms involved in controlling *MYCN* gene expression in liver cancer, with a focus on inflammation-mediated signal transduction and microRNA-associated post-transcriptional regulation. We concluded that dynamic *MYCN* gene expression is an integrated consequence of multiple signals in the tumor microenvironment, including tumor growth-promoting signals, lipid desaturation-mediated endoplasmic reticulum stress adaptation signals, and tumor suppressive miRNAs, making it a potential predictive biomarker of tumor stemness and plasticity. Therefore, understanding and tracing the dynamic changes and functions of *MYCN* gene expression will shed light on the origin of liver tumorigenesis at the cellular level and the development of novel therapeutic and diagnostic strategies for liver cancer treatment.

## Introduction

Liver cancer, mostly hepatocellular carcinoma (HCC), is a highly lethal cancer (>600,000 deaths per year worldwide) in which approximately 10% of patients survive the first 5 years after diagnosis (1). Liver cancer is recognized as an inflammation-related cancer, since more than 90% of HCC cases arise in the context of chronic liver injury and unresolved inflammatory microenvironment due to viral infection, alcohol consumption, or high-fat diet (HFD) hypernutrition (2–4). Advances in antiviral therapy have reduced the risk of developing hepatitis B virus- and hepatitis C virus-related HCC (5, 6). In contrast, non-alcoholic steatohepatitis (NASH) which is characterized by obesity-associated inflammation has attracted much attention, and is believed that it will soon be the leading etiology of HCC (7). Notably, mice fed with HFD alone did not develop liver injury and tumorigenesis. However, hyperresponsivity to lipopolysaccharide and endoplasmic reticulum (ER) stress were observed in fatty liver that contributed to the progression of NASH and HCC (8, 9). This suggested that a non-genomic mechanism was involved in the control of cellular responses such as adaptation to inflammatory stresses during hepatic tumorigenesis. In this line, whereas tumor initiation depends on somatic mutations, the mechanisms underlying tumor promotion are likely to involve epigenetic factors and environmental factors extrinsic to the cancer cell (10).

MYCN is a canonical proto-oncogene basic helix-loop-helix transcription factor that is mainly restricted to the migrating neural crest (11) and governs cell growth and differentiation during embryonic stages (12). Amplification of the *MYCN* locus was first observed in human neuroblastoma (13). *MYCN* amplification is observed in about 20% of neuroblastoma and represents one of the strongest clinical predictors of poor prognosis (14). *MYCN* amplicons are either organized as extrachromosomal double minutes or as homogeneously stained regions in addition to the single copy of *MYCN* on the short arm of chromosome 2, retained at 2p24, in neuroblastoma cells and other solid tumor cells (15). Notably, the *MYCN* gene is located in a non-fragile region of 2.8 Mbp between two common fragile sites, FRA2Ctel and FRA2Ccen, located at 2p24.3 and 2p24.4, respectively (16). A study by Blumrich and colleagues suggested that *MYCN* amplicons might arise from extra rounds of replication of unbroken DNA secondary structures that accumulate at FRA2C (16). Recent clinical studies have reported increased gene expression of *MYCN* in liver tumor tissues (17, 18). However, according to The Cancer Genome Atlas (TCGA) database, nine of the 371 HCC patients (2.4%) with upregulated *MYCN* mRNA expression but not the seven patients (1.9%) with *MYCN* amplification had a dramatically worse prognosis (**Figure S1A**). Data mining using the Cancer Cell Line Encyclopedia (CCLE) database identified a total of 65 *MYCN* mutations, but none of them was detected in HCC cell lines irrespective of their corresponding mRNA abundance (**Table S1**). This highlights the existence of non-genomic mechanisms potentially responsible for *MYCN* overexpression in liver cancer. Notably, data mining in TCGA showed that the expression of *MYCN* in human HCC was not correlated with that of *c-MYC*, another MYC family membranes known to be crucial for liver cancer maintenance (19) and oncogenic reprogramming of terminally differentiated hepatocytes into liver cancer stem cells (CSCs) (20) (**Figure S1B**). In addition, *MYCN* gene expression but not *c-MYC* gene expression was significantly correlated with the liver CSC marker *EpCAM* gene expression (**Figure S1B**). These data highlight the possibility that *MYCN* gene expression is restricted in CSC-like cells and serves as a more sensitive biomarker than *c-MYC* gene expression for the detection of tumor stemness during liver tumorigenesis. Here, we reviewed the role of dynamic *MYCN* gene expression in liver homeostasis, regeneration, and tumorigenesis, and discussed the potential non-genomic mechanisms involved in controlling *MYCN* gene expression in liver cancer, focusing on inflammation-mediated signal transduction and microRNA-associated (miRNA)-post-transcriptional regulation.

## MYCN Gene Expression in Liver Homeostasis, Regeneration, and Tumorigenesis

Single-cell RNA sequencing provided a comprehensive view of *MYCN* gene expression in both human and mouse livers (21, 22). Under steady-state conditions, the expression of *MYCN* gene is low in hepatocytes (**Figure S1C**) (21). *MYCN* gene expression in the liver is significantly zonated, which is predominantly induced in the pericentral cells and progressively decreases along the liver lobule towards periportal cells (**Figure S1D**) (22). Metabolic liver zonation requires a Wnt/β-catenin signaling gradient (23). In the uninjured liver, diffusible Wnt ligands produced by the pericentral endothelial cells activate β-catenin signaling-induced target genes such as *Axin2* and maintain a population of proliferating and self-renewing cells, surrounding the central vein, that contribute to homeostatic hepatocyte renewal (24). Wnt/β-catenin signaling is critical for organ development, homeostasis, and regeneration through governing stem cell pluripotency (25). During neocortical development, *MYCN* is a direct downstream target of the Wnt/β-catenin pathway and promotes neuronal fate commitment (26). Therefore, the basal expression of *MYCN* gene in the liver is a likely consequence of the activation of Wnt/β-catenin signaling during liver homeostasis.

Cap Analysis of Gene Expression(CAGE)-based transcriptional profiling of isolated primary mouse hepatocytes revealed that low level of *MYCN* gene expression was detected at 2 h and peaked at 48 h after 70% partial hepatectomy (**Figure S1E**) (27). Liver regeneration is a coordinated multistep process that is largely dependent on the re-entry of differentiated adult hepatocytes into the cell cycle and proliferation (28). In response to loss of hepatic tissue, hepatocyte DNA synthesis peaks at around 24 h, accompanied by the induction of gene expression of growth-regulated and cell-cycle-regulated genes at around 48 h (29). It is possible that the induction of *MYCN* gene expression is a mitogenic response of hepatocytes during liver regeneration. Indeed, a major direct mitogen of hepatocytes, the epidermal growth factor (EGF), stimulated *MYCN* gene expression in neuroblastoma cells *via* the recruitment of the transcription factor Sp1 to the *MYCN* promoter region (30).

Transcriptome profiling of frozen human liver tissues using microarray showed that *MYCN* gene expression was low in healthy livers, cirrhotic livers, and adjacent non-tumorous liver tissue, while it was dramatically increased in tumor tissues (17). Project HOPE (High-tech Omics-based Patient Evaluation), a clinical study aiming to provide multi-omics data of cancer patients, showed the upregulation of *MYCN* gene expression in tumor tissues compared to normal tissues in 22% of recruited HCC patients (18). Our previous cohort studies in Japan (n = 102) and Europe (n = 50) confirmed an increase in *MYCN* gene expression in HCC tumor regions as compared to non-tumor regions (17). Importantly, in a long-term (>10 years) follow-up study, *MYCN* gene expression in HCC tumors was significantly higher in patients with recurrence than in those without recurrence and was positively correlated with the *de novo* recurrence of HCC with a single tumor but not with multiple tumors (17). HCC recurrence at approximately 1–2 years after resection was considered to be mainly due to *de novo* carcinogenesis of liver CSCs or tumor-initiating cells (31). *MYCN* gene expression in HCC was positively correlated with the expression of liver CSC markers and Wnt/β-catenin signaling markers, suggesting that MYCN expression is restricted to CSC-like HCC (17). Consistently, MYCN expression marked an EpCAM^+^ CSC-like subpopulation, which was selectively depleted by acyclic retinoid (ACR), a promising chemopreventive agent against the recurrence of HCC after curative treatment (17, 32). EpCAM is a well-characterized liver CSC marker and is a direct transcriptional target of Wnt/β-catenin signaling (33). Similar to liver homeostasis, the restricted MYCN expression in liver CSCs is probably related to the activation of Wnt/β-catenin signaling. Furthermore, four out of six liver biopsies of HCC patients (66.7%) who had received 8 weeks of high-dose ACR treatment (600 mg/day), but not low-dose ACR treatment (300 mg/day), after definitive treatment showed decreased *MYCN* gene expression (< 0.5-fold) (17). In line with this, clinical studies showed that administration of ACR at 600 mg/day, but not 300 mg/day, reduced HCC recurrence after curative treatment (34). Collectively, MYCN expression marked CSC-like subpopulations in heterogeneous HCC and served as a potential therapeutic target and prognostic marker for HCC.

## Regulation of Mycn Gene Expression by Tissue Repair Signals in the Inflammatory Microenvironment of Liver Cancer

Activation of inflammatory signal transduction in the tumor microenvironment is strongly linked to tumor initiation and progression based on two mechanisms: tissue repair and stress adaptation. Obesity-associated production of inflammatory cytokines, such as interleukin-6 (IL-6) and tumor necrosis factor-α (TNFα), induce repeated liver injury and compensatory proliferation, which might lead to aberrant stabilization and activation of “repair signals” such as signal transducer and activator of transcription 3 (STAT3)-dependent oncogenic signaling pathways and initiation and progression of HCC (35–37). The involvement of hyperactivated IL-6-STAT3 signaling axis as a driver oncogenic mechanism in promoting cell proliferation and suppressing antitumor immune response in the background of tumor microenvironment has been reported in several cancers (38). STAT3 directly mediates the initiation of *MYCN* transcription in neuroblastoma cells (39). Inhibition of STAT3 with antisense oligonucleotide or pharmacological inhibitors reduced *MYCN* gene expression and decreased neuroblastoma tumorigenicity in preclinical mouse models (39, 40). During early hepatocarcinogenesis, STAT3 activated by paracrine IL-6 produced by inflammatory cells, might directly bind to the promoter and upregulate the gene and protein expression of CD133, a well-defined liver CSC marker representing a specialized subpopulation of highly tumorigenic cells with high MYCN expression (17, 41, 42). Inhibition of STAT3 with sorafenib, the first-line recommended therapy for patients with advanced HCC, decreased CD133 levels and suppressed *in vivo* tumorigenicity by eradicating the liver tumor microenvironment (41). Of note, a recent proteomics-based pathway analysis showed that sorafenib inactivated downstream signaling of MYCN in HCC cells (43). In addition, growth factors such as EGF induced by inflammatory cytokines contribute to the upregulation of *MYCN* gene expression in an inflammatory microenvironment (30). Nerve growth factor (NGF) is expressed by hepatocytes during fibrotic liver injury (44). In *MYCN*-amplified neuroblastoma cells, NGF suppressed *MYCN* gene expression through mitogen-activated protein kinase signaling pathways (45). In contrast, a global transcriptome analysis showed reduced *MYCN* gene expression in NGF-deprived sympathetic neurons (46). It is unclear whether NGF directly regulates *MYCN* gene expression in normal livers and HCC cells.

## Regulation of MYCN Gene Expression by Lipid Desaturation-Mediated Stress Adaptation Signals in the Inflammatory Microenvironment of Liver Cancer

The cell membrane serves as the barrier between life and death for individual cells and the first line of defense in response to environmental stress. In addition to their function as energy storage sources or as building blocks of membranes, membrane lipids have attracted much attention as biologically active molecules. They regulate the formation of membrane assembly of signal complexes by either binding to cognate receptors or recruiting proteins from the cytosol and coordinating signal transduction (47). Membrane lipids are highly diverse in chemical structures, varying in the desaturation and chain elongation of fatty acyl chains, backbones (such as glycerol, sphingoid base, and cholesterol), and head group substituents. Changes in membrane lipid composition affect membrane physical properties, as observed in mammalian cells in response to environmental stimuli (47). For example, macrophages rapidly reprogram their lipid metabolism, especially *de novo* cholesterol biosynthesis (48, 49) and desaturated fatty acid biosynthesis (50), for appropriate inflammatory and host defense functions in response to diverse inflammatory signals. Under inflammatory conditions, the fatty acid synthetic enzyme fatty acid synthase, reshapes macrophage lipid homeostasis for the assembly of cholesterol-dependent inflammatory signals such as Rho GTPase at the plasma membrane (51). In contrast, lipid desaturases such as stearoyl-CoA desaturase (SCD1) and fatty acid desaturase (FADS) were induced to inhibit the inflammatory responses through the production of anti-inflammatory omega-3 polyunsaturated fatty acids or disruption of membrane signaling complexes associated with lipid rafts, also known as membrane microdomains, which are enriched with saturated sphingolipids and cholesterol (52). Importantly, lipid reprograming, especially the upregulation of unsaturated fatty acids, has recently been recognized as a critical feature of stem cell maintenance under both physiological and abnormal conditions (53, 54). Our previous proteome and metabolome analyses demonstrated that high MYCN expression in liver CSCs was characterized by increased expression of lipid desaturases such as SCD1 and FADS and elevated levels of monounsaturated fatty acids such as palmitoleic acid and oleic acid in comparison to non-CSC HCC cells (55, 56). In addition to the upstream regulatory role of MYCN in lipid metabolism reprograming of cancer cells [reviewed in (57)], inhibition of lipid desaturation using both genetic and pharmacological approaches against SCD1 reduced *MYCN* gene expression and selectively suppressed the proliferation of high *MYCN*-expressing HCC cells, suggesting a direct regulatory role of lipid desaturation on *MYCN* transcription (56). Genome-wide transcriptome analysis using RNA-seq showed that ER stress-related signaling pathways were regulated upon siRNA knock-down of *SCD1* but not *MYCN* in high MYCN-expressing HCC cells (56). Further, mechanistic studies showed that inhibition of lipid desaturation resulted in activation of ER stress signaling pathways, such as the expression of the transcription suppressor, cyclic AMP-dependent transcription factor 3 (ATF3), which reversibly regulates *MYCN* gene expression in high MYCN-expressing CSC-like HCC cells, CSC-rich spheroids, and in clinical HCC tissues (56).

**Figure 1:**
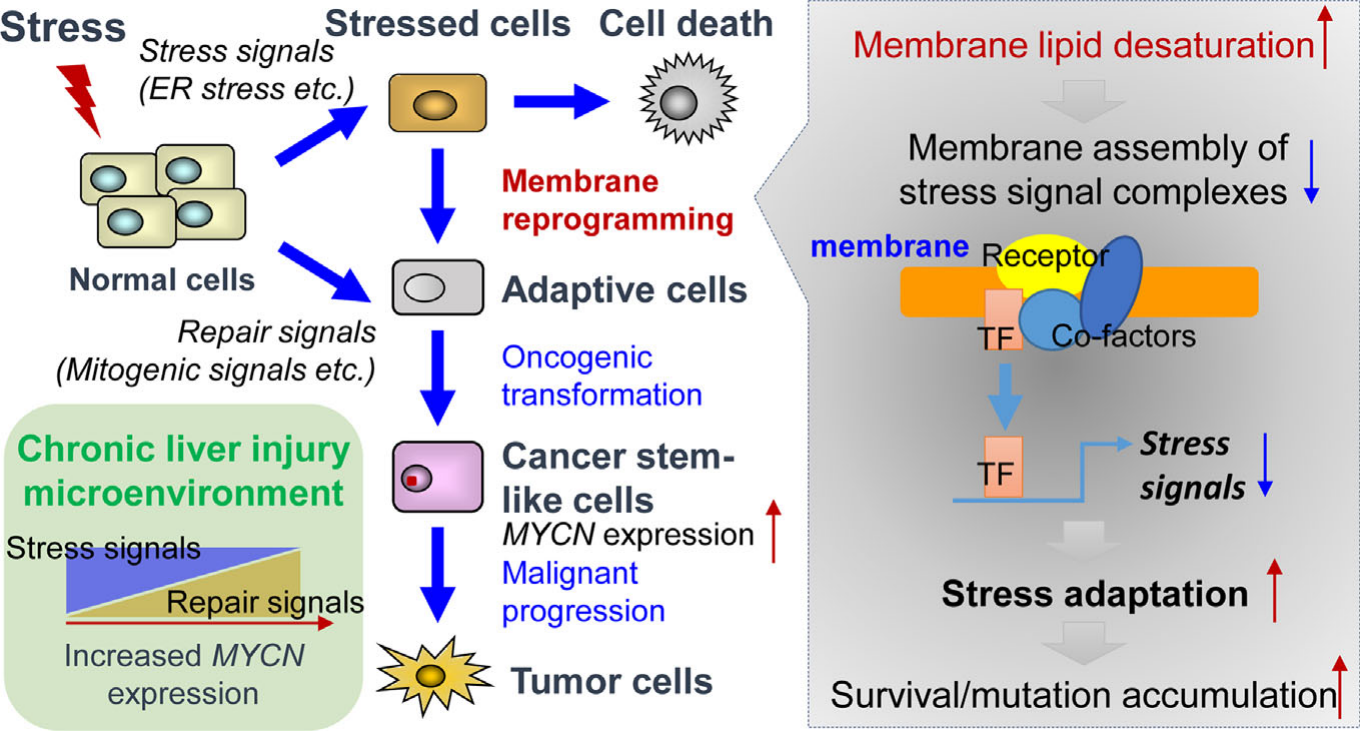
Tissue repair and stress adaptation signal-based control of *MYCN* gene expression in the inflammatory microenvironment of liver cancer. Under lipid-rich inflammatory conditions, inflammatory cytokines-induced chronic injury leads to the activation of repair signals such as mitogenic signals resulting in compensatory proliferation, thereby triggering downstream *MYCN* gene expression. In contrast, lipid desaturation-mediated membrane reprogramming reduces or counteracts the formation of membrane assembly of stress signal complexes and enables the CSCs to survive and evade ER stress-induced apoptosis/differentiation, which leads to the rescue of *MYCN* gene expression and initiates tumorigenesis by accumulation of mutations in long-living CSCs.

ER stress response, also known as unfolded protein response (UPR), is activated as a cell-defensive mechanism triggered by multiple stress factors and plays a critical role in the switch between cell survival and cell death. Evading ER stress-induced apoptosis and differentiation is critical for the maintenance of long-living and self-renewing stem cells under both normal and malignant conditions (58–60). Therefore, modulation of ER stress-induced loss of stemness represents a potential therapeutic strategy for cancers and chronic inflammatory diseases (61, 62). In line with this, pharmacological targeting of SCD1 achieved remarkable therapeutic outcomes in glioblastoma and liver cancer by triggering ER stress-mediated apoptosis and differentiation of CSCs (63, 64). Mechanistically, enhanced levels of unsaturated fatty acids in CSCs could suppress ER stress by preventing saturated fatty acid-induced calcium accumulation, oxidative stress, or detrimental stiffening of the ER and plasma membrane (65–68). Collectively, under lipid-rich inflammatory conditions, both repair signals and stress adaptation signals contribute to the upregulation of *MYCN* gene expression (**Figure 1**). Inflammatory cytokine-induced chronic injury leads to the activation of repair signals, which triggers downstream *MYCN* gene expression and compensatory proliferation. In contrast, lipid desaturation-mediated membrane reprogramming reduces or counteracts the formation of membrane assembly of stress signal complexes and enables CSCs to survive and evade ER stress-induced apoptosis/differentiation. We propose that the stress adaption mechanism in long-living CSC-like cells contributes to tumorigenesis such as through accumulation of mutations in the survived cells, which is accompanied by the increase of *MYCN* gene expression.

## Post-Transcriptional Control of MYCN Gene Expression by Mirnas in Liver Cancer

miRNAs are evolutionarily conserved small non-coding RNAs of approximately 22 nucleotides in length that modulate gene expression by complementary base pairing with the 3’-untranslated regions (3’-UTRs) of messenger RNAs [reviewed in (69)]. An essential feature of miRNA-based gene regulation is that a single miRNA can recognize numerous mRNAs and, conversely, a target mRNA can be recognized by several miRNAs. A large number of studies have reported the key role of these posttranscriptional regulators in the control of various cellular processes and human diseases (70). In cancer, aberrant expression of miRNAs has been well described and is associated with the deregulation of critical genes involved in tumor progression (71). Indeed, cancer-related miRNAs can act as oncogenes (called oncomirs) or tumor-suppressors, depending on their targets, and promote or negatively influence tumor growth, invasion, and/or drug resistance, respectively (72). Specific miRNA profiles have been identified in neuroblastoma, which reflect different subtypes of tumors and correlate with the advancement of the disease or its prognosis [reviewed in (73)]. In this malignancy, numerous *MYCN*-targeting miRNAs have been identified. Loss of miR-34a at chromosome band 1p36, a region frequently deleted due to loss of heterozygosity in neuroblastoma cells (74), is associated with *MYCN* amplification and promotion of tumor aggressiveness (75). Thus far, several additional miRNA/MYCN regulatory axes have been characterized. In a model of *MYCN*-amplified neuroblastoma cells, experimental overexpression of miR-101 and let-7e induced a decrease in MYCN protein levels and inhibited cell growth *via* the direct regulation of MYCN (76, 77). In another interesting study by Neviani and colleagues, the tumor-suppressor miR-186 was detected in natural killer cell-derived exosomes, which exhibited cytotoxicity against neuroblastoma cells with high *MYCN* levels (78). The authors showed that *MYCN* expression was directly inhibited by miR-186. In addition, the targeted delivery of miR-186 to *MYCN*-amplified neuroblastoma cells or natural killer cells resulted in significant tumor growth inhibition. A recent study based on the modeling of miRNA-mRNA interactions identified a regulatory loop between *MYCN* and miR-204 in neuroblastoma cells (79). The authors showed that miR-204 directly targeted *MYCN* mRNA and decreased its protein levels. In contrast, MYCN was able to bind to the promoter of miR-204 and inhibit the expression of the miRNA. Remarkably, the capability of MYCN to activate the expression of critical oncomirs, such as miR-221, miR-9, or the miR-17-92 cluster, has also been observed in neuroblastoma cells and other types of solid tumor cells (80).

**Figure 2:**
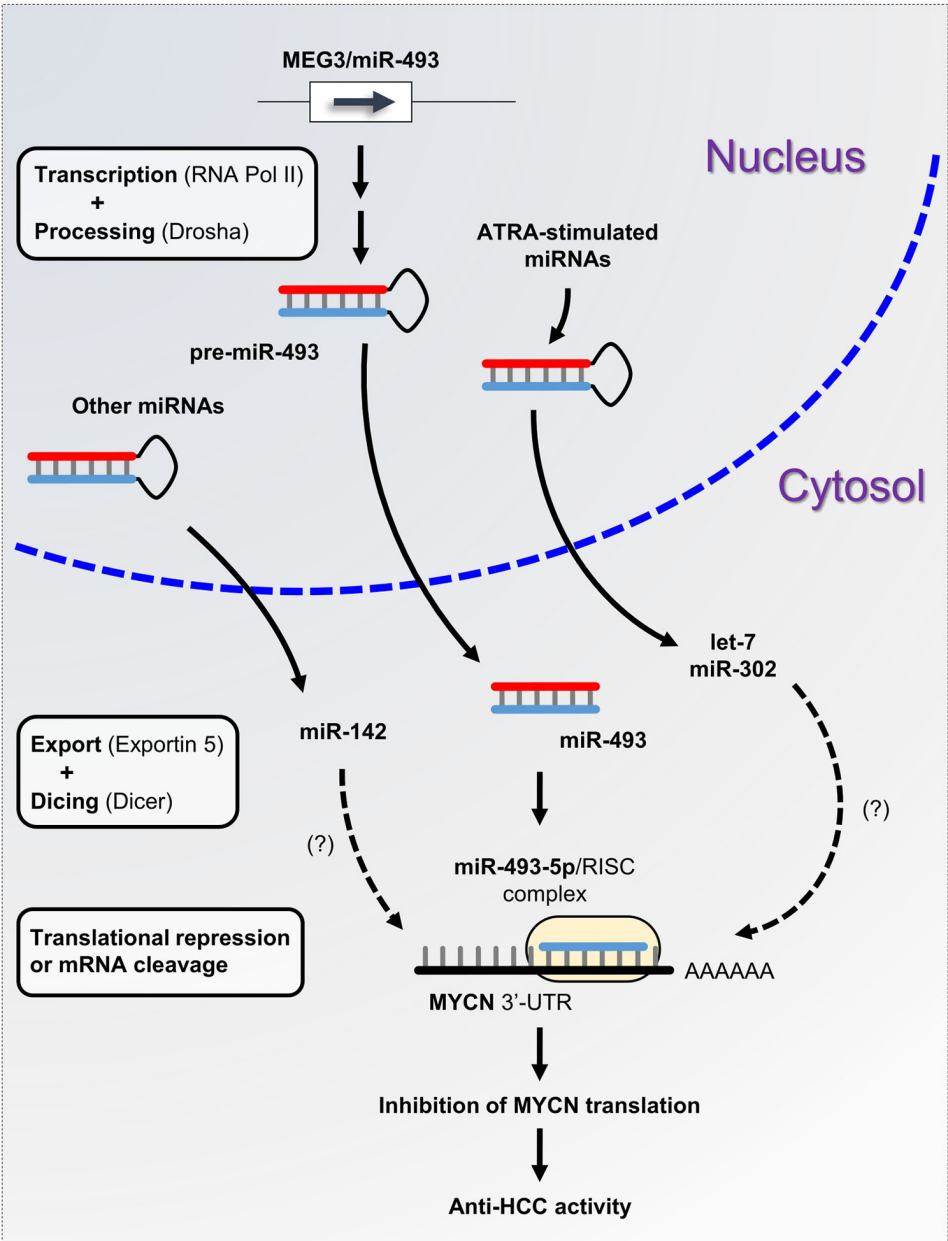
miRNA-based control of *MYCN* gene expression in liver cancer. miRNA biogenesis is a multistep process. Following transcription by RNA polymerase II, primary precursor miRNAs (pri-miRNAs) are cleaved into precursor miRNAs (pre-miRNAs) by the RNase III enzyme Drosha and exported out of the nucleus to produce mature miRNAs. Subsequently, mature miRNAs are loaded onto the RNA-induced silencing complex (RISC) and directed to the 3’-UTR of target mRNAs. Here, we propose the miRNA/MYCN regulatory network model, in which the tumor-suppressor miR-493-5p and the ATRA-stimulated miRNAs modulate *MYCN* expression and impede HCC progression.

A plethora of studies have described the functional interconnection between miRNAs and MYCN in neuroblastoma. However, little is known about the miRNAs involved in the posttranscriptional regulation of *MYCN* in liver cancer. The aberrant expression of miRNAs is a typical hallmark of hepatocarcinogenesis and tumor progression (81). We previously demonstrated that maternally expressed 3 (MEG3)-derived miR-493-5p tumor-suppressor was epigenetically silenced by CpG hypermethylation in HCC cells and tumor tissues from patients (82). Experimental overexpression of miR-493-5p promoted an anti-cancer response by inhibiting HCC cell growth and invasion, in part, through the negative regulation of insulin-like growth factor 2 (IGF2) and the IGF2-derived intronic oncomir miR-483-3p. More recently, our group highlighted *MYCN* as another major target of miR-493-5p using global gene expression analysis of liver cancer cells with restored expression of miR-493-5p (83). More precisely, real-time qPCR data showed an inverse and significant correlation between miR-493-5p and *MYCN* expression levels in the tumors of patients with advanced HCC. A dual-luciferase reporter activity assay validated miR-493-5p-mediated inhibition of *MYCN via* the targeting of two distinct regions in the *MYCN* 3’-UTR (**Figure 2**). To the best of our knowledge, no additional miRNA interacting directly with *MYCN* mRNA has been described in liver cancer thus far. However, in a study based on big data mining and connectivity map analysis, Xiong et al. uncovered the existence of a potential hsa_circRNA_104515/hsa-miR-142-5p/MYCN regulatory axis in HCC (84). In agreement with this finding, we found that TargetScanHuman predicted an exact consequential pairing of the *MYCN* 3’-UTR with positions 2-8 (7mer-m8) of mature miR-142-5p (**Figure S1F**). Interestingly, two reports described downregulation of miR-142-5p in liver cancer cells and showed that forced expression of miR-142-5p inhibited HCC cell growth and invasion (85, 86). Taken together, these data strongly suggest the tumor-suppressive role of miR-142-5p through post-transcriptional control of *MYCN* and its therapeutic potential in liver cancer. Finally, recent studies showed that all-*trans* retinoic acid (ATRA), which is an isomer of retinoic acid, was able to modulate the expression of more than 300 miRNAs and inhibit the growth of various types of tumor cells (87). Among the miRNAs upregulated after ATRA treatment, miR-34a-5p, miR-103a-3p, miR-200b/c-3p, miR-302-3p, and members of the let-7 family appeared appealing given their potential ability to target the *MYCN* 3’-UTR as predicted by TargetScanHuman 7.2 (**Figure S1F**). While the tumor-suppressor feature of the let-7 family members has been well-documented, further investigations will be required to evaluate the beneficial role of ATRA-stimulated miRNAs in HCC, since some of these miRNAs may also exhibit oncogenic activity.

## Conclusions

Mature hepatocytes exhibit remarkable plasticity by direct dedifferentiation into an undifferentiated state in the tumor microenvironment, which are believed to represent the cells of origin for liver cancer (88). Since any cell has the potential to become a CSC, the stemness of liver CSCs could be considered as a dynamic state that can be acquired rather than a cell intrinsic property of specialized existing cells [reviewed in (89)]. *MYCN* gene is overexpressed in restricted cell populations such as EpCAM^+^ CSCs in liver cancer, regardless of DNA amplification and mutation. Dynamic *MYCN* gene expression is an integrated consequence of multiple signals in the tumor microenvironment, including tumor stemness/growth-promoting signals such as Wnt/β-catenin and IL-6-STAT3 signaling, lipid desaturation-mediated ER stress adaptation signals, and tumor suppressive miRNAs. We propose that *MYCN* gene expression might represent a potential predictive biomarker of tumor stemness and plasticity. Hence, understanding and tracing the dynamic changes and functions of *MYCN* gene expression during hepatic tumorigenesis will shed light on the origin of liver tumorigenesis at the cellular level and the development of novel therapeutic and diagnostic strategies for HCC treatment.

## Author Contributions

X-YQ and LG performed the literature search and wrote the manuscript. All authors contributed to the article and approved the submitted version.

## Funding

This work was supported by grants from the Ministry of Education, Culture, Sports, Science and Technology of Japan’s Grant-in-Aid for Scientific Research (C), JP20K07349 (to X-YQ), and JP20K07604 (to LG) and RIKEN Incentive Research Projects (to X-YQ and LG).

## Conflict of Interest

The authors declare that the research was conducted in the absence of any commercial or financial relationships that could be construed as a potential conflict of interest.

## Supplementary Material

Supplementary Figure 1 Supporting data of *MYCN* gene expression in the liver. **(A)** Overall survival Kaplan-Meier estimate of HCC patients with *MYCN* overexpression (left) or amplification (right) according to TCGA database (TCGA, PanCancer Atlas). **(B)** Correlation between *MYCN* gene expression and *c-MYC* gene expression (left), *MYCN* gene expression and *EpCAM* gene expression (middle), and *c-MYC* gene expression and *EpCAM* gene expression (right) in human HCC according to TCGA database (TCGA, PanCancer Atlas). **(C)** *MYCN* gene expression pattern in human liver visualized using a web interface (http://human-liver-cell-atlas.ie-freiburg.mpg.de/), which is based on the single cell RNA-seq data published in (21). **(D)** *Mycn* gene expression in mouse liver zonation according to the single cell RNA-seq data (GSE84498) published in (22). **(E)** *Mycn* gene expression in mouse primary hepatocyte isolated at 2, 30, 48 h or 1 w after partial hepatectomy and at 2 h from sham control during liver regeneration. The data was obtained from the CAGE-based transcriptome data published in **Table S1** in (27). **(F)** Prediction of miR-142-5p, miR-34a-5p, miR-103a-3p, miR-200b/c-3p, miR-302-3p, and members of the let-7 family targeting MYCN 3’-UTR according to TargetScanHuman (http://www.targetscan.org, release 7.2).
