# Supplementary figures and images for: Non-Genomic Control of Dynamic MYCN Gene Expression in Liver Cancer
Source: Front Oncol. 2021 Apr 16;10:618515. doi: 10.3389/fonc.2020.618515 (PMC8085327; doi:10.3389/fonc.2020.618515)

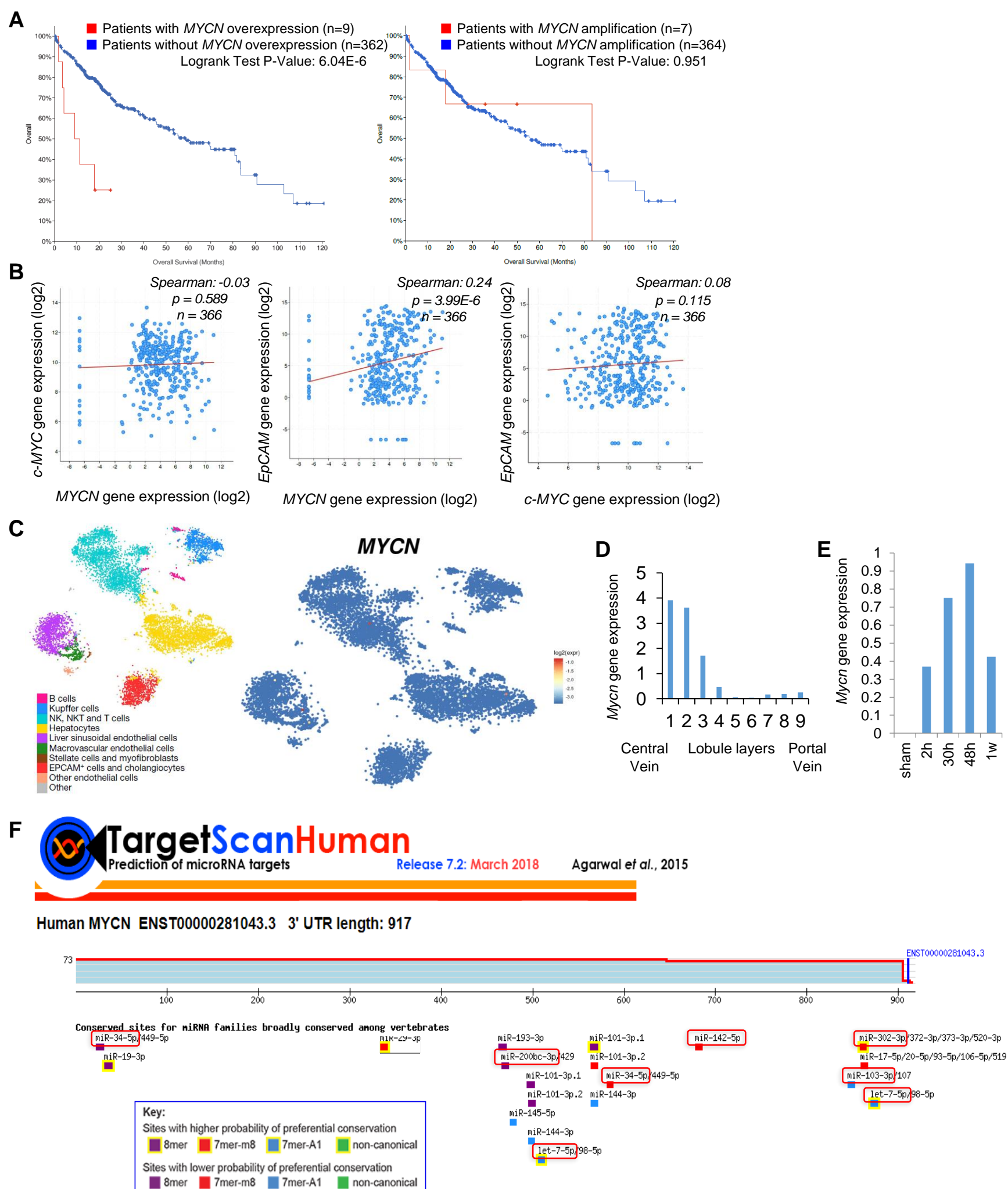

**Fig. S1**

Supplement: Supplementary Figure 1 — Supporting data of MYCN gene expression in the liver. (A) Overall survival Kaplan-Meier estimate of HCC patients with MYCN overexpression (left) or amplification (right) according to TCGA database (TCGA, PanCancer Atlas). (B) Correlation between MYCN gene expression and c-MYC gene expression (left), MYCN gene expression and EpCAM gene expression (middle), and c-MYC gene expression and EpCAM gene expression (right) in human HCC according to TCGA database (TCGA, PanCancer Atlas). (C) MYCN gene expression pattern in human liver visualized using a web interface (http://human-liver-cell-atlas.ie-freiburg.mpg.de/), which is based on the single cell RNA-seq data published in (21). (D) Mycn gene expression in mouse liver zonation according to the single cell RNA-seq data (GSE84498) published in (22). (E) Mycn gene expression in mouse primary hepatocyte isolated at 2, 30, 48 h or 1 w after partial hepatectomy and at 2 h from sham control during liver regeneration. The data was obtained from the CAGE-based transcriptome data published in Table S1 in (27). (F) Prediction of miR-142-5p, miR-34a-5p, miR-103a-3p, miR-200b/c-3p, miR-302-3p, and members of the let-7 family targeting MYCN 3’-UTR according to TargetScanHuman (http://www.targetscan.org, release 7.2). [file Image_1.pdf]
